# Pyrene removal from contaminated soils by modified Fenton oxidation using iron nano particles

**DOI:** 10.1186/2052-336X-11-17

**Published:** 2013-07-16

**Authors:** Sahand Jorfi, Abbas Rezaee, Ghasem-ali Moheb-ali, Nemat alah Jaafarzadeh

**Affiliations:** 1 Department of Environmental Health Engineering, School of Medical Sciences, Tarbiat Modares University, Tehran, Iran; 2 Biotechnology Research Center, Research Institute of Petroleum Industry, Tehran, Iran; 3 Department of Environmental Health Engineering, School of Public Health, Environmental Technology Research Center, Ahwaz Jondishapour University of Medical Sciences, Ahwaz, Iran

**Keywords:** Soil pollution, Pyrene, Modified fenton oxidation, Iron nano oxide, Chelating agent

## Abstract

**Background:**

The problems related to conventional Fenton oxidation, including low pH required and production of considerable amounts of sludge have led researchers to investigate chelating agents which might improve the operating range of pH and the use of nano iron particle to reduce the excess sludge. The pyrene removal from contaminated soils by modified Fenton oxidation at neutral pH was defined as the main objective of the current study.

**Methods:**

Varying concentrations of H_2_O_2_ (0-500 mM) and iron nano oxide (0-60 mM), reaction times of 0.5-24 hours and variety of chelating agents including sodium pyrophosphate, sodium citrate, ethylene diamine tetraacetic, fulvic and humic acid were all investigated at pyrene concentration levels of 100 – 500 mg/kg.

**Results:**

By applying the following conditions (H_2_O_2_ concentration of 300 mM, iron nano oxide of 30 mM, sodium pyrophosphate as chelating agent, pH 3 and reaction time of 6 hours) the pyrene removal efficiency at an initial concentration of 100 mg/kg was found to be 99%. As a result, the pyrene concentration was reduced from 100 to 93 mg/kg once the above optimum conditions are met.

**Conclusions:**

In this research, the modified Fenton oxidation using iron nano oxide at optimum conditions is introduced as an efficient alternative method in lab scale for chemical remediation or pre-treatment of soils contaminated by pyrene at neutral pH.

## Introduction

Polycyclic Aomatic Hydrocarbons (PAHs) are persistent contaminants resulting from natural or anthropogenic processes. Industrial processes, petroleum product spillages and incomplete combustion of fossil fuels have led to PAHs accumulation during past decades [1]. Environmental contamination to PAHs and specially soil pollution near the gas stations or petrochemical industries is verified in several reports. The concerning characteristics of PAHs is their hydrophobic nature which increases the toxicity to the human and environment [2]. Therefore, they are easily adsorbed to soil matrix and make tight bindings with Soil Organic Matter (SOMs) [3].

Pyrene is a four-ring PAH with low biodegradability and high persistency in environment, which is considered as a priority pollutant by US EPA because of its carcinogenic and mutagenic effects [4-6]. Various methods including solvent extraction, phytoremediation, elecrokinetic and photocatalytic remediation, thermal destruction, chemical remediation and bioremediation are evaluated and studied for PAHs removal and transformations. Chemical remediation and bioremediation are preferred because of their superior advantages. However, bioremediation is restricted due to being time consuming, degradation of only one or few contaminants with a specified strain, the possible toxicity of pollutant to the bacterial strains and high toxicity and persistency of majority of hydrocarbons [7,8].

Since the chemical oxidation approaches act vigorously and rapidly and are not sensitive to the type of pollutants, they can be considered as a suitable alternative for bioremediation or can be used as pre or post-treatment in a sequencing system with bioremediation [9]. The required time for remediation can be significantly reduced using Advanced Oxidation Processes (AOPs) like Fenton oxidation. In conventional Fenton oxidation, H_2_O_2_ reacts with soluble Fe ^2+^ and yields hydroxyl radical (OH^•^) and Fe^3+^ (equation 1). Yap et al. [10] demonstrated the efficiency of Fenton oxidation for pyrene, anthracene and phenanthrene removal in soil with kinetic constants ranging from 2.5 × 10^-1^ h^-1^ to 3.9 × 10^-1^ h^-1^[10]. Also, Kulic et al (2006) reported that Fenton oxidation of PAHs obey pseudo first order reactions that an increase in Fenton reagents led to improvement of removal efficiency [11]. Hydroxyl radical is a non selective oxidant with redox potential of 2.8, which is able to break down PAHs and many other organic persistent pollutants. In conventional Fenton reaction, the regeneration of Fe ^2+^ from produced Fe^3+^ is necessary (equation 2), which needs acidic pH to prevent Fe^3+^precipitation as iron hydroxide [12].

(1) $$Fe^{2+}+H_{2}O_{2}\to Fe^{3+}OH^{*}+OH^{‒}$$

(2) $$Fe^{3+}+H_{2}O_{2}\to Fe^{2+}+H^{+}+HO_{2}{}^{*}$$

This low pH can adversely affect the natural soil systems and change its characteristics, and on the other hand is not adaptable with the possible following bioprocess [13,14]. Chelating agents (CAs) are added to conventional Fenton reaction to enhance the catalytic activity of soluble iron and prevent iron loss due to its precipitation or binding to hydrophobic portions of soil organic matters at neutral pH. This modification is called modified Fenton oxidation [15]. The chelating agents with their multidentate features and cyclic structure can bind metal ions to form heterocyclic rings. In addition to organic chelating agents like ethylene diamine tetra acetic acid (EDTA), oxalic acid (OA), sodium citrate (SC), fulvic acid (FA), humic acid (HA), cathecol and acetic acid, modified Fenton oxidation with inorganic CAs is also studied. Venny et al. [16] considered sodium pyrophosphate as a chelating agent for PAHs removal in contaminated soils in neutral pH [16]. The most important advantages of inorganic CAs include the less scavenging effects to hydroxyl radicals and not increasing the total carbon content of the soil during treatment. Pyrophosphate is successfully used for Fenton reactions at neutral pH and the phosphate ions are a supplementary nutrient source for soil matrix and microbial metabolism [16]. Another difficulty in Fenton reaction is rapid dissociation of H_2_O_2_ with Fe ^2+^, which usually hinders effective application of Fenton oxidation. Therefore, Fe ^3+^ is used instead of Fe^2+^ to prevent the short half life of H_2_O_2_ in slurry soil [17]. Iron nano oxides have been frequently studied for remediation of contaminated sites in recent years, because of their advantages including high reactivity, less amounts of excess sludge and secondary metabolites, effective degradation of contaminants and destruction of a wide range of pollutants. Rusevova et al. [18] studied nano-sized magnetic iron oxides as catalysts for heterogeneous Fenton-like reactions [18]. Also Grieger et al. [19] studied the environmental benefits and the risks of iron nano particle for in situ remediation [19]. In the current study, the application of iron nano oxides in a modified Fenton oxidation using organic and inorganic CAs for pyrene removal was defined as the main objective to determine the feasibility of the process at natural pH and also possible superiority of iron nano oxides over iron salts.

## Materials and Methods

### Chemicals

**Figure 1:**
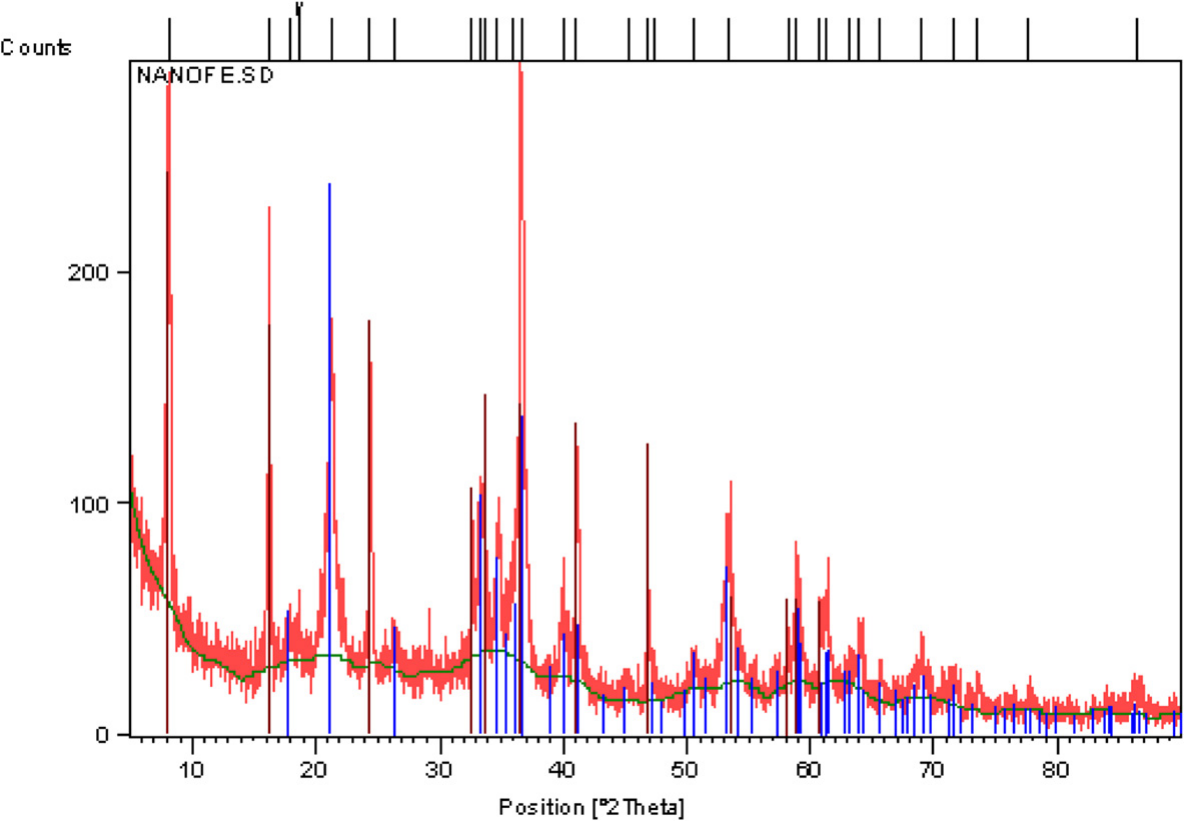
The XRD analysis of iron nano oxides.

pyrene (96%) for preparation of the stock solution of 1 g/L and all solvents including n-hexane, methanol and acetone were of analytical grade and purchased from [Merck Company]. Hydrogen peroxide (30%), H_2_SO_4_ (98%), HCl, NaOH, EDTA, sodium citrate, sodium pyrophosphate, HgCl_2_, FeSO_4_.7H_2_O were also purchased from Merck Company. Humic and fulvic acid were supplied by Fluka Company. Iron nano oxides (Both Fe_3_O_4_ and Fe_2_O_3_ were confirmed by XRD analysis (Figure 1) were prepared from Zist Shimi Azma Corporation.

### Soil preparation

The soil was derived from an oil industrial zone in south of Iran. The Soil samples were collected from the upper layer (0–40 cm) by soil cores, air-dried, and passed through a 2-mm sieve. Then, the sieved soils were homogenized by shaking, washed three times with acetone, autoclaved and stored in plastic containers at 4°C. The resulting samples were spiked with a solution of 100 mg/kg pyrene dissolved in n-hexane, shacked and left in hood for 24 h, till the n-hexane evaporated. The stock solution of pyrene was placed on a shaker for 1 minute before spiking. For simulation of an old soil contamination, a portion of spiked soil sample was aged for one year and then used in experiments [20]. The soil was analyzed for physical and chemical characteristics by Tarbiat Modares University Engineering Soils Testing Lab, Iran (Table 1). The chemical composition of the soil sample was determined byX Ray Flourescence (XRF) analysis.

**Table 1:** The soil characteristics

**Characteristic**	**Value (%)**	**Characteristic**	**Value (%)**
Soil type	Silty sand	L.O.I	10.7
Sand	74.3	Na_2_O	1.944
Clay	1.02	MgO	2.046
Silt	24.68	Al_2_O_3_	17.095
Specific surface area (m^2^/g)	5.39	SiO_2_	54.797
Moisture content (%)	6.78	P_2_O_5_	0.202
Fe_2_O_3_	0.4155	K_2_O	3.163
Cu	0.158	CaO	8.842
Sr	0.047	TiO_2_	0.569
Zr	0.019	-	-

The soil classification was found to be silty sand with 1.02% clay. Among all of its constituents, Natural Organic Matter (NOM) and native iron were important regarding Fenton oxidation. NOM plays an important role in scavenging OH^•^ radical needed for PAH oxidation and iron content plays a catalytic role for decomposition of H_2_O_2_. The primary concentration of combustible or volatile content (as an indirect indicator of NOM) and Fe oxides in the soil sample were 107, and 4.155 g/kg, respectively.

### Modified Fenton oxidation of pyrene

Following soil spiking and preparation, the contaminated samples were subjected to modified Fenton oxidation in the slurry phase. A 5 g soil sample containing 100 mg/kg pyrene was placed in a 100-mL glass flask with screw cap and then 15 mL of distilled water containing 0.2% HgCl_2_ (W/V)( to inhibit the potential biological activity) was added to make the slurry phase (water : soil , 3:1 w/w) [21].

The experimental procedure was designed according to one factor at the time experimental design. In the first run, the iron nano oxide at concentration of 5 mM was added to each flask and mixed for 10 minute to insure the homogenous distribution of nano oxides in the soil medium. The pH of the mixture was adjusted to 3 using 2 N HCl.

The reaction was initiated by gradual (five steps during the whole reaction time) addition of varying concentrations of 30% H_2_O_2_ (0-500 mM; 0.155, 0.31, .0.47, 0.62, 0.78 mL 30% H_2_O_2_ containing 51, 102, 153, 204 and 255 mg H_2_O_2_/15 mL, respectively) to each flask. The homogeneity was provided by shaking the samples at 180 rpm (IKM 4000 ci, Germany) at 30 ± 0.5 °C. After 30 minutes, the reaction was terminated with an addition of 1 mL of 1.5 M sodium thiosulphate. In the second run, the effect of varying concentrations of iron nano oxide (5-60 mM; 14.625, 29.25, 58.5, 87.75, 117, 146.25 and 175.5 mg Iron nano powder/15 mL) were studied at pH 3, contact time of 30 minutes and the optimum H_2_O_2_ concentration derived from the first run.

In the third run, varying contact times (0.5- 24 h) were studied at optimum H_2_O_2_ and Fe^3+^ concentrations at pH 3. Then, according to the optimum conditions obtained from the previous runs, the pyrene removal efficiency was investigated at pH 7 in the presence of sodium pyrophosphate (as inorganic CA) and EDTA, FA, HA and SC (as organic CA).

In order to assess the effect of each chelating agent on removal efficiency, non parametric Kruskal Wallis test was used for statistical analysis. Kruskal Wallis test was selected because we had to deal with a limited number of variables (including chelating agent) and repetition (three times). Finally, higher pyrene concentration and the aged soil sample were investigated according to the optimum conditions at pH 7 to simulate the process efficiency for field applications with aged and highly contaminated soils by PAHs. It is worth noting that all experiments were conducted in triplicate.

### Pyrene extraction and quantification

The residual pyrene in the soil sample was extracted using an ultrasound device according to EPA method 3550B. Breifly; the sample was placed into a special tube filled with a mixture of n-hexane and acetone (1:1 v/v). After the extraction, it was centrifuged for 10 min at 6000 rpm and filtered by PTFE. A portion of the filtered solution was removed for final analysis [22]. The concentration of pyrene was determined by gas chromatography (GC) system (Chrompack CP 9001) equipped with a flame ionization detector (FID) using (HP-5) capillary column (30 m, 0.32 mm i.d. and 0.2 μm film thickness). Nitrogen was used as carrier gas at a rate of 2 mL/min. The temperature program was as follows: the column temperature was held at 120°C for 1 min and then raised to 240°C at a rate of 20°C/min, again held for 1 min. The injector and detector temperatures were set at 250 and 300°C, respectively.

## Results

### Optimum concentration of H_2_O_2_ and iron nano oxide

**Figure 2:**
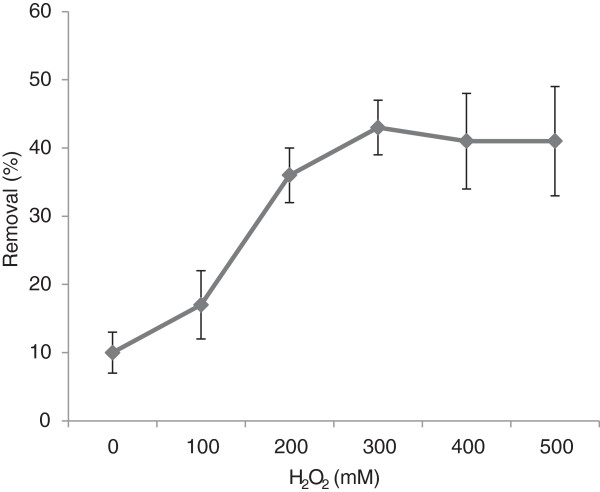
**The pyrene removal efficiency in varying H**_**2**_**O**_**2 **_**concentrations of 0-500 mM, reaction time of 30 min, pH 3 and iron nano oxides concentration of 5 mM.**

The effect of varying H_2_O_2_ concentrations on pyrene removal efficiency (at an initial concentration of 100 mg/kg) was investigated and presented in Figure 2. As H_2_O_2_ concenteration rises, the removal of pyrene is increased and finally leveled off. The maximum removal efficiency of 43% was observed at the H_2_O_2_ concentration of 300 mM, as can be seen in Figure 2. Therefore, H_2_O_2_ concentration of 300 mM was considered as the optimum value for further expriments.

**Figure 3:**
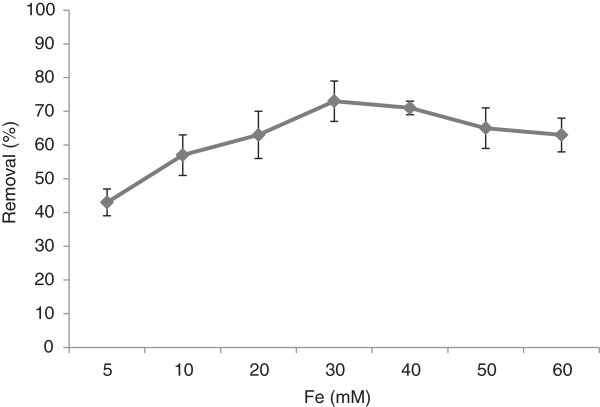
**The pyrene removal efficiency versus varying Fe concentrations in optimum H**_**2**_**O**_**2 **_**concentration of 300 mM, reaction time of 30 min and pH 3.**

In the next stage, the effect of various concentrations of iron nano oxide on the Fenton oxidation of pyrene was investigated at optimum H_2_O_2_ concentration of 300 mM. The removal efficiency was increased with an increase in Fe^3+^ dosage up to 30 mM, and then decreased at higher concentrations (Figure 3). The pyrene removal reached 73% at the optimum concentration levels of H_2_O_2_ and iron nano oxide. As can be deducted from Figure 3, the iron nano oxide concentration of 30 mM and H_2_O_2_/Fe molar ratio of 10 were taken as the optimal values.

### The effect of contact time on pyrene removal

**Figure 4:**
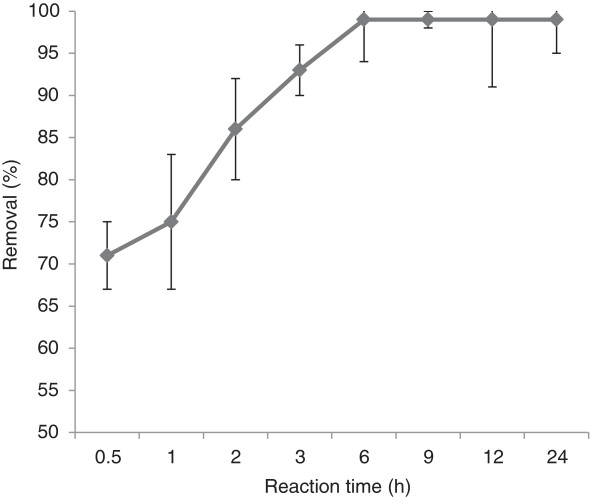
**Effect of contact time on pyrene removal by Fenton oxidation in optimum H**_**2**_**O**_**2 **_**and Fe concentrations and pH 3.**

The effect of reaction time on pyrene removal at optimum H_2_O_2_ and Fe^3+^ concentration was investigated. According to Figure 4, reaction time positively affected the removal efficiency and the pyrene removal of 99% was achieved after 6 hours of contact time. After all, the soil contaminated by an initial pyrene concentration of 100 mg/kg was completely removed under the following conditions: H_2_O_2_ concentration of 300 mM, Fe^3+^ concentration of 30 mM, pH 3 and the reaction time of 6 h.

### Effect of chelating agent on oxidation efficiency at neutral pH

**Figure 5:**
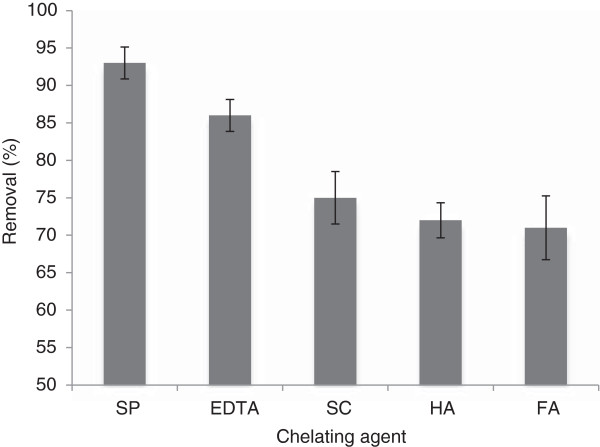
**The pyrene removal efficiency for different CAs in pH 7, H**_**2**_**O**_**2 **_**concentration of 300 mM, Fe**^**3+ **^**concentration of 30 mM and Contact time of 6 h.**

Due to the operational problems with regard to Fenton oxidation at acidic pH in full scale applications such as soil matrix destruction, damages to native plant, and release of toxic metal, the ability of a CA to keep the iron catalyst in the solution at natural pH is highly considered for removal of organic pollutants in soil via Fenton oxidation. The pyrene removal in the presence of sodium pyrophosphate (as an inorganic CA) and EDTA, FA, HA and SC (as organic CA) at H_2_O_2_/Fe^3+^ molar ratio of 10, pH 7 and the contact time of 6 h was investigated at the pyrene initial concentration of 100 mg/kg. The removal efficiency was found to be 93% for sodium pyrophosphate and 86%, 75%, 72% and 71% for EDTA, SC, HA and FA ,respectively (Figure 5). The effect of a number of chelating agents on pyrene removal efficiency from contaminated soil at optimum conditions was significant (P < 0.019) according to non parametric Kruskal Wallis test. The results of statistical analysis are presented in Table 2.

**Table 2:** The statistical analysis of each chelating agent application (according to non parametric Kruskal Wallis test)

**Group**	**Mean removal ± Std**	**P Value**
FA	70 ± 4.26	P < 0.019
HA	72 ± 2.34	
SC	75 ± 3.49	
EDTA	86 ± 2.14	
SP	93 ± 2.12	

**Figure 6:**
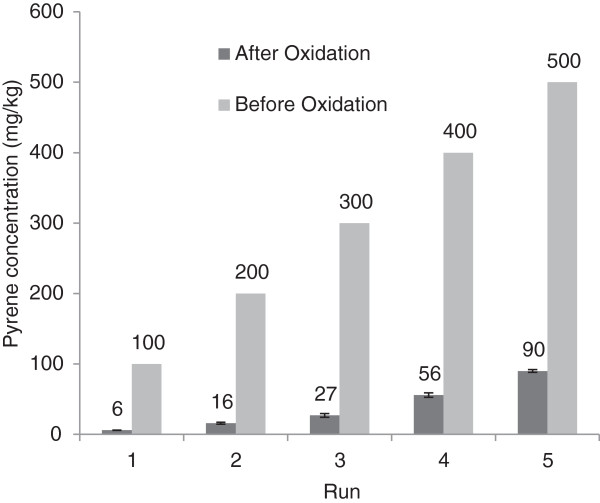
**The pyrene concentration variations in optimum modified Fenton oxidation including pH 7, H**_**2**_**O**_**2**_ **= 300 mM, Fe**^**3+**^ **= 30 mM, reaction time of 6 h and pyrophosphate.**

Regarding the removal efficiency of 93% (obtained at pH 7, CA of pyrophosphate, H_2_O_2_ concentration of 300 mM, Fe^3+^ concentration of 30 mM and the contact time of 6 h), higher concentrations of pyrene (100 -500 mg/kg) were investigated to simulate actual conditions in the presence of other hydrocarbons with more cumulative concentration. By applying the optimum conditions of the modified Fenton oxidation, the residual pyrene was decreased to 6, 16, 27, 56 and 90 mg/kg at initial concentrations of 100, 200, 300, 400 and 500 mg/kg, respectively (Figure 6).

### The effect of pollution aging on oxidation efficiency

In order to investigate the efficiency of modified Fenton oxidation according to the optimum conditions obtained in the current study, a soil sample spiked by a pyrene solution of100 mg/kg and aged up to one year, and also a real contaminated soil sample with different hydrocarbons were both subjected to the modified Fenton oxidation. After the extraction, the amounts of pyrene extracted in the aged and real soil sample were found to be 86.8 and 67 mg/kg, respectively. However, the residual pyrene in the soil sample with aged contamination was 7.8 mg/kg providing the total removal of 91% for pyrene in the contaminated soil was obtained. The total residual pyrene in the aged soil sample was 21 mg/kg (13.2 mg/kg, non-extracted; 7.8 mg/kg, remaining after modified Fenton oxidation). The residual pyrene for the real soil sample was found to be 38 mg/kg (removal of 43%).

## Discussion

The optimum dosages of H_2_O_2_, iron catalyst and consequently H_2_O_2_/Fe ratio are critical operational issues in Fenton oxidation. In various studies, a range of concentrations and molar ratios are introduced as optimum conditions. The principal reasons for this variation can arise from the type of pollutant, contaminant concentration, reaction time, age of pollution and the inorganic and organic characteristics of soil [10]. Since Fenton oxidation obeys pseudo first order kinetics, increase in reagent dosages effectively enhances the process efficiency [10].

In the current study, pyrene removal increased at higher H_2_O_2_ concentrations, but no considerable improvement was observed at H_2_O_2_ concentrations beyond 300 mM. Hydroxyl radicals produced by reaction 1 is the main factor for the destruction of persistent organic contaminants. In order to have continuous production of hydroxyl radicals, Fe^3+^ produced from reaction 1 should be reduced to Fe^2+^ by an additional reaction (reaction 2). The pyrene removal is restricted, when sufficient iron catalyst and excess amounts of H_2_O_2_ are present. Consequently, H_2_O_2_ in excess consumes considerable amounts of Fe^2+^ leading to a shortage of Fe^2+^ in the reaction. This could explain why the removal efficiency falls at higher H_2_O_2_ concentrations (i.e., >300 mM).

The optimum concentration of iron catalyst is also very important. In this study, iron nano oxide at 30 mM was determined to be the best dosage. At higher values, pyrene removal efficiency was increased, but alongside an observed increase in the amounts of excess sludge, as a negative side effect of Fenton oxidation. Applying other iron types including zero valent iron nano particles or iron nano oxides instead of soluble iron (Fe^2+^) to decrease the excess sludge production, is one of the most important modifications in the conventional Fenton reaction. The excess sludge level is considerably decreased during Fenton oxidation when iron nano particles are applied as the catalyst for H_2_O_2_ dissociation [23]. On the other hand, in presence of excess amounts of Fe^2+^, H_2_O_2_ is either turned into water via side reactions (instead of radical production) or acts as a radical scavenger [24]. Kulic et al (2006) claimed that increasing the Fenton reagents led to a 15% improvement in removal of PAHs [11]. Nam et al. [25] reported that an increase in the Fe^2+^/H_2_O_2_ ratio favored only hydrophobic compounds like three, four and five ring PAHs. Since pyrene is a four-ring PAH, the removal enhancement at higher reagent concentrations can be justified [25].

In a similar study as reported here, Sun et al. [26] reported that the optimum H_2_O_2_ and Fe^2+^ concentrations they found were 200 and 20 mM, respectively; with a H_2_O_2_/Fe^2+^ molar ratio of 10) [26]. In further related studies, H_2_O_2_/Fe^2+^ molar ratios of 5 to 25 were reported as the optimum values which is in agreement with the current study finding [27]. The low pH (= 3) needed for conventional Fenton oxidation is a major difficulty in full scale applications. Generally, Low pH is an unsuitable alternative compared to bioremediation, especially in soil remediation. It enhances heavy metal mobility and changes the ecosystem of the treated soil. The chelating agents are used at neutral pH to overcome this limitation. In the current study, pyrene removal of 93% was obtained by the usage of SP as an inorganic CA at pH 7, which was more than the maximum removal of 86% obtained for EDTA amongst organic CAs. It is believed that EDTA is superior to other organic CAs like FA, HA and SC because of its strong five membered structure yielding the tight binding EDTA-iron complex which is highly stable even at neutral soil pH [16]. Hence, inorganic CAs cannot compete with hydroxyl radicals and therefore cannot increase the TOC content of the soil. However, they can enhance the pyrene removal through modified Fenton oxidation more than that of organic CAs [16].

Venny et al. [16] reported that the flouranthene removal by a modified Fenton reaction using SP (as CA) was 78%, which is lower than the values obtained in the current study [16]. The differences in the reaction time, the type of PAH, soil and oxidation conditions maybe explain this result. Also, Kanel et al reported 85% removal for phenanthrene in a Fenton like oxidation at neutral pH after 3 h contact time [12].

Generally, hydrocarbon removal decreases along with contamination aging, which is confirmed by the current study findings. Pollution aging leads to migration of pyrene from easily accessible to difficult sites (sequestration), which reduces the molecule oxidation. Sequestration is a natural process happening in soil which reduces the chemical and biological remediation efficiency of hydrophobic organic pollutants [28]. Fenton oxidation can alter the combination state of hydrophobic organic compounds and enhance their desorption [27].

In the current work, pyrene removal in freshly contaminated soil was observed to be greater than that of the soil sample with aged pollution providing the same experimental conditions are applied to all samples. As a matter of fact, pyrene removal in the real soil sample contaminated with a pyrene concentration of 67 mg/kg was relatively low (only 43%). This low removal efficiency can be attributed to the presence of other hydrocarbons scavenging hydroxyl radicals and also the contamination aging. This finding is in agreement with previous work by Sun and Yan [26].

The removal efficiency for an initial pyrene concentration of 40 mg/kg was reported to decrease from 88.9% in freshly contaminated soil to 74.7% in soil aged for 30 days [26]. Consequently, the full scale applications of modified Fenton oxidation for PAHs removal, requires higher reagent concentrations compared to lab scale amounts.

For simulation of actual conditions with cumulative concentrations of hydrocarbons, higher pyrene concentrations were studied. The pyrene removal efficiency for initial concentrations of 400 and 500 mg/kg was 86% and 82%, respectively. These removal values would be even less in actual conditions with aging of the pollution. Also, when the removal efficiency is considerably high value, it will breakdown a large portion of pyrene or metabolizes it, leading to relatively easy degradation via biological processes. The pyrene removal at optimum conditions obtained in this study was 90% for an initial pyrene concentration of 300 mg/kg.

## Conclusions

According to the findings of this study and the considerable benefits obtained from the use of iron nanoparticles, including less excess sludge and a faster reaction rate, it can be concluded that the modified Fenton oxidation using iron nano oxides at optimum concentrations of H_2_O_2_ and Fe^3+^ and also in the presence of sodium pyrophosphate as inorganic CA was an efficient alternative in lab scale experiments for remediation or pre-treatment of soils contaminated by pyrene at neutral pH. Therefore, in the future, more studies in full scale applications along with economic analysis with other technologies should be completed to assess the process feasibility and viability for field applications.

## Abbreviations

AOPs: Advanced oxidation processes; CA: Chelating agent; EDTA: Ethylene diamine tetra acetic acid; FA: Fulvic acid; HA: Humic acid; H2O2: Hydrogen peroxide; OA: Oxalic acid; PAH: Polycyclic aromatic hydrocarbon; SC: Sodium citrate; SOMs: Soil organic matters; USEPA: United stated Environmental Protection Agency; XRD: X-ray Diffraction; XRF: X-ray fluorescence.

## Competing interests

The authors declare that they have no competing interests.

## Authors’ contributions

This paper was extracted from a PhD thesis and Dr AR, Dr NJ and Dr GM were designed and directed the study as supervisor and consulter and participated in its coordination and helped to draft the manuscript. SJ was the student who did the experiments and wrote the paper. All authors read and approved the final manuscript.

## References

[B1] Rezaei Kalantary R Badkoubi A Mohseni-Bandpi A Esrafili A Jorfi S Emad Dehghanifard E Baneshi M Modification of PAHs Biodegradation with Humic Compounds, Soil and Sediment Contamination Soil and sediment contamination: An International Journal 2013 22 185 198 10.1080/15320383.2013.722139

[B2] Nadarajah N Van HJ Pannu J Singh A Enhanced transformation of polycyclic aromatic hydrocarbon using a combined Fenton’s reagent, microbial treatment and surfactants Applied Microbial Biotechnology 2002 59 540 544 10.1007/s00253-002-1073-x 12172623

[B3] Mohsenzadeh F Chehregani Rad A Akbari M Evaluation of oil removal efficiency and enzymatic activity in some fungal strains for bioremediation of petroleum-polluted soils Iranian Journal of Environmental Health Sciences & Engineering 2012 9 26 10.1186/1735-2746-9-26 PMC3561093 23369665

[B4] Srikanth RM Naresh B Leel T Prashanthi M Madhusudhan N Dhanasri G Devi P Biodegradation of phenanthrene with biosurfactant production by a new strain of *Brevibacillus sp* Bioresour Technol 2010 101 7980 7983 10.1016/j.biortech.2010.04.054 20627713

[B5] Chung MK Hu R Cheung KC Wong MH Pollutants in Hong Kong soils: polycyclic aromatic hydrocarbons Chemosphere 2006 67 464 473 17109918 10.1016/j.chemosphere.2006.09.062

[B6] Zhou W Zhu L Efficiency of surfactant-enhanced desorption for contaminated soils depending on the component characteristics of soil-surfactant- PAHs system Environ. Pollution 2007 147 66 73 10.1016/j.envpol.2006.08.018 17070632

[B7] Watts R Stanton P Howsawkeng J Teel A Mineralization of a sorbed polycyclic aromatic hydrocarbon in two soils using catalyzed hydrogen peroxide Water Res 2002 36 4283 4292 10.1016/S0043-1354(02)00142-2 12420933

[B8] Ferrarese E Andreottola G Oprea L Remediation of PAH-contaminated sediments by chemical oxidation J Hazard Mater 2008 152 128 139 10.1016/j.jhazmat.2007.06.080 17689010

[B9] Lin Q Yingxu C Wang Z Wang Y Study on the possibility of hydrogen peroxide pretreatment and plant system to remediate soil pollution Chemosphere 2004 57 1439 1447 10.1016/j.chemosphere.2004.08.070 15519388

[B10] Yap C Gan S Ng H Fenton based remediation of polycyclic aromatic hydrocarbons-contaminated soils Chemosphere 2011 83 1414 1430 10.1016/j.chemosphere.2011.01.026 21316731

[B11] Kulik N Goi A Trapido M Tuhkanen T Degradation of polycyclic aromatic hydrocarbons by combined chemical pre-oxidation and bioremediation in creosote contaminated soil J Environ Manage 2006 78 382 391 10.1016/j.jenvman.2005.05.005 16154683

[B12] Kanel SR Neppolian B Choi H Yang JW Heterogenous catalytic oxidation of phenanthrene by hydrogen peroxide in soil slurry: kinetic, mechanism, and implication Soil Sediment Contamination 2003 12 101 117

[B13] Valderrama C Alessandri R Aunola T Cortina JL Gamisans X Tuhkanen T Oxidation by Fenton’s reagent combined with biological treatment applied to a creosote-contaminated soil J Hazard Mater 2009 166 594 602 10.1016/j.jhazmat.2008.11.108 19135785

[B14] Yeh CK Hsu C Chiu C Huang K Reaction efficiencies and rate constants for the goethite-catalysed Fenton-like reaction of NAPL-form aromatic hydrocarbons and chloroethylenes Journal of Hazardous Material 2008 151 562 569 10.1016/j.jhazmat.2007.06.014 17673366

[B15] Georgi A Schierz A Trommler U Horwitz C Collins T Kopinke F Humic acid modified Fenton reagent for enhancement of the working pH range Applied Catalyst B-Environment 2007 72 26 36 10.1016/j.apcatb.2006.10.009

[B16] Venny Gan S Ng HK Inorganic chelated modified-Fenton treatment of polycyclic aromatic hydrocarbon (PAH)-contaminated soils Chem Eng J 2012 180 1 8 10.1016/j.scitotenv.2011.12.053 22285087

[B17] Kanel SR Neppolian B Jung H Choi H Comparative removal of polycyclic aromatic hydrocarbons using iron oxide and hydrogen peroxide in soil slurries Environ Eng Sci 2004 21 741 751 10.1089/ees.2004.21.741

[B18] Rusevova K Kopinke F Georgi A Nano-sized magnetic iron oxides as catalysts for heterogeneous Fenton-like reactions—Influence of Fe(II)/Fe(III) ratio on catalytic performance J Hazard Mater 2012 241–242 433 440 23098995 10.1016/j.jhazmat.2012.09.068

[B19] Grieger K Fjordbøge A Hartmann N Eriksson E Bjerg P Baun A Environmental benefits and risks of zero-valent iron nanoparticles (nZVI) for *in situ* remediation: Risk mitigation or trade-off J Contam Hydrol 2010 118 165 183 10.1016/j.jconhyd.2010.07.011 20813426

[B20] Khan E Wirojanagud W Sermsal N Effects of Iron type in Fenton reaction on mineralization and biodegradability enhancement of hazardous organic compounds J Hazard Mater 2009 161 1024 1034 10.1016/j.jhazmat.2008.04.049 18502575

[B21] Bogan B Trbovic V Effect of sequestration on PAH degradability with Fenton’s reagent: roles of total organic carbon, humin, and soil porosity J Hazard Mater 2003 100 285 300 10.1016/S0304-3894(03)00134-1 12835029

[B22] PAHs Extraction Methodhttp://www.trincoll.edu/~henderso/textfi~1/416%20notes/3550b.pdf

[B23] Liao C Chung T Chen W Kuo S Treatment of pentachlorophenol-contaminated soil using nano-scale zero-valent iron with hydrogen peroxide Journal of Molecular Catalysis A: Chemical 2007 265 189 194 10.1016/j.molcata.2006.09.050

[B24] Choi K Lee W Enhanced degradation of trichloroethylene in nano-scale zero-valent iron Fenton system with Cu(II) J Hazard Mater 2012 211–212 146 153 22079185 10.1016/j.jhazmat.2011.10.056

[B25] Nam K Rodriguez W Kukor JJ Enhanced biodegradation of polycyclic aromatic hydrocarbons by biodegradation combined with a modified Fenton reaction Chemosphere 2001 45 11 20 10.1016/S0045-6535(01)00051-0 11572585

[B26] Sun H Yan Q Influence of pyrene combination state in soils on its treatment efficiency by Fenton oxidation J Environ Manage 2008 88 556 563 10.1016/j.jenvman.2007.03.031 17517464

[B27] Moon B Park Y Park K Fenton oxidation of Orange II by pre-reduction using nanoscale zero-valent iron Desalination 2011 268 249 252 10.1016/j.desal.2010.10.036

[B28] Jonsson S Persson Y Frankki S Bavel B Lundstedt S Haglund P Tysklind M Degradation of polycyclic aromatic hydrocarbons (PAHs) in contaminated soils by Fenton’s reagent: a multivariate evaluation of the importance of soil characteristics and PAH properties J Hazard Mater 2007 149 86 96 10.1016/j.jhazmat.2007.03.057 17513044

